# Percutaneous native renal biopsy adequacy: a successful interdepartmental quality improvement activity

**DOI:** 10.1186/s40697-015-0043-z

**Published:** 2015-03-13

**Authors:** Laurette Geldenhuys, Peter Nicholson, Namita Sinha, Angela Dini, Steve Doucette, Talal Alfaadhel, Valerie Keough, Michael West

**Affiliations:** Department of Pathology and Laboratory Medicine, Dalhousie University, Halifax, Nova Scotia Canada; Department of Community Health and Epidemiology, Dalhousie University, Halifax, Nova Scotia Canada; Department of Medicine, Dalhousie University, Halifax, Nova Scotia Canada; Department of Diagnostic Imaging, Dalhousie University, Halifax, Nova Scotia Canada

**Keywords:** Native and allograft renal biopsy adequacy

## Abstract

**Background:**

An adequate renal biopsy is essential for diagnosis and treatment of medical renal disease.

**Objective:**

We evaluated two initiatives to improve adequacy of renal biopsy samples at our centre.

**Design:**

Retrospective determination of renal biopsy adequacy.

**Setting:**

Queen Elizabeth II Health Sciences Centre.

**Patients:**

Patients undergoing medical renal biopsies.

**Measurements:**

Renal biopsy adequacy.

**Methods:**

The first initiative was to restrict the performance of biopsies to a smaller group of radiologists and to include a comment on biopsy adequacy in every pathology report. The second initiative was to introduce on-site adequacy assessment by a medical laboratory technologist. Native renal and allograft biopsy adequacies were calculated for three periods: 1) baseline, October 2005 to September 2006; 2) after implementation of the first initiative, January 2007 to September 2011; and 3) after implementation of the second initiative, October 2011 to September 2012. A subset of native renal biopsies was examined to determine if there was a relationship between adequacy and number of passes.

**Results:**

The percentages of adequate native renal biopsies during the first, second, and third periods were 31%, 72% and 90%, respectively. This represents a significant increase (40%, p < 0.0001) in adequacy following the first initiative, and another significant increase (18%, p = 0.0003) following the second initiative. The percentages of adequate renal allograft biopsies during the first, second, and third periods were 75%, 56% and 69%, respectively. These changes in adequacy were not statistically significant. In the subset of native renal biopsies examined, a biopsy comprising more than three cores was not associated with increase in adequacy.

**Limitations:**

The most important limitation is the lack of generally accepted and applied adequacy criteria limiting generalizability of our findings.

**Conclusions:**

Restricting the performance of biopsies to subspecialist operators, including an adequacy statement in the renal biopsy report and on-site adequacy assessment were effective in significantly improving native renal biopsy adequacy. This improvement appeared unrelated to an increase in the number of passes taken with a biopsy needle. Neither initiative improved the low adequacy of allograft biopsies.

## Abrégé

**Contexte:**

L’importance que joue la biopsie percutanée dans le diagnostic et le traitement des maladies rénales est un fait bien connu. Un spécimen adéquat est nécessaire pour une interprétation correcte des résultats. La surveillance de la qualité de biopsie reste une pratique importante de contrôle de la qualité.

**Objectif de l’étude:**

Deux initiatives visant une meilleure qualité des échantillons rénaux pour la biopsie ont été évaluées à notre centre.

**Type d’étude:**

Détermination rétrospective de la qualité de la biopsie rénale.

**Contexte:**

Le Queen Elizabeth II Health Sciences Centre.

**Patients:**

Patients subissant des biopsies rénales.

**Méthodes:**

La première initiative visait à restreindre la réalisation des biopsies à un petit groupe de radiologues et d’ajouter un commentaire sur la qualité de la biopsie dans chaque rapport de pathologie. La deuxième initiative visait à implanter l’évaluation de cette qualité sur place, par l’intermédiaire d’un technicien de laboratoire médical. La qualité des biopsies de reins natifs et d’allogreffes ont été calculées en trois temps : 1) mesure de référence: d’octobre 2005 à septembre 2006; 2) après l’implantation de la première initiative, de janvier 2007 à septembre 2011; 3) après l’implémentation de la deuxième initiative, d’octobre 2011 à septembre 2012. Un sous-ensemble de biopsies effectuées sur des reins natifs a été examiné afin de déterminer s’il existait un lien entre la qualité et le nombre de ponctions.

**Résultats:**

Les pourcentages d’une qualité adéquate des biopsies de reins natifs des trois phases étaient de 31%, 72% et 90%, respectivement. On remarque des hausses de la qualité des biopsies importantes après la première initiative (40%, p < 0,0001) et après la deuxième (18%, p = 0,0003). Les pourcentages d’une qualité adéquate des biopsies d’allogreffes rénales des trois étapes étaient de 75%, 56% et 69%, respectivement; ces changements n’étaient pas statistiquement significatifs. Dans le sous-ensemble des biopsies de reins natifs, celles de plus de trois échantillons n’étaient pas associées avec une hausse de la qualité.

**Limites de l’étude:**

Parmi les limites de l’étude, on compte les données lacunaires concernant : les complications postopératoires; l’analyse détaillée des caractéristiques des radiologistes avant et après la première intervention; certaines données permettant de mesurer l’effet de la deuxième intervention indépendamment de son lien avec la première intervention; un consensus sur les critères de la qualité des biopsies de reins natifs et d’allogreffes rénales à des fins de comparaisons entre les différentes institutions, et qui s’est répercuté sur la généralisabilité de nos résultats (provenant d’un seul centre) à d’autres établissements. Le manque de puissance statistique a limité la possibilité de détecter certaines différences entre les sous-groupes.

**Conclusions:**

La restriction de la réalisation des biopsies par une sous-spécialité, l’ajout d’un commentaire sur la qualité de la biopsie rénale dans le rapport, et l’évaluation sur place de la qualité ont eu l’effet d’améliorer de façon significative la proportion de biopsies rénales natives avec une qualité adéquate. Cette amélioration ne semble pas liée à une hausse du nombre de ponctions faites avec une aiguille à biopsie. Aucune des initiatives n’a amélioré la basse qualité des biopsies d’allogreffes.

## What was known before

An adequate renal biopsy specimen is required for accurate interpretation.

## What this adds

Restricting the performance of biopsies to subspecialist operators, including an adequacy statement in the renal biopsy report and on-site adequacy assessment were effective in significantly improving native renal biopsy adequacy. The increase in adequacy was not related to an increase in number of passes with a biopsy needle to a statistically significant extent.

## Background

The importance of the percutaneous biopsy in the diagnosis and treatment of renal diseases is well-established [1]. The renal biopsy procedure that yields clinically relevant information is a multidisciplinary task that depends upon close cooperation between nephrologists, radiologists, technologists and pathologists [2]. An adequate specimen is required for accurate interpretation [3]. Inadequate specimens result in delays in diagnosis and treatment, and increased health-care costs in addition to the risk of a repeated biopsy procedure. Because of the impact on patient care, monitoring of biopsy adequacy is an important quality assurance activity.

Historically, the definition of an adequate biopsy has been based upon either: 1) the opinion of the pathologist that there is sufficient tissue to make a diagnosis, or 2) fixed criteria based upon the number of glomeruli and vessels present in the specimen. In the case of allograft biopsies, the numerical criteria established in the Banff 97 guidelines have been generally accepted and applied [4,5]. Despite the statistical demonstration of minimum glomerular counts needed to exclude and accurately stage focal renal disease [3,6], no numerical adequacy criteria for native renal biopsies have found wide acceptance.

At our centre locally defined numerical criteria were applied as part of a clinical audit of renal biopsy adequacy. It was retrospectively determined that the percentage of adequate native renal biopsies for the period between October, 2005, and September, 2006, was 31%, which was much lower than adequacy reported in the literature. Specific reasons for this unusually low adequacy could not be identified, and even allowing for the difficulties of comparison caused by the lack of established adequacy criteria in the literature, the local adequacy was felt to be sufficiently low to require intervention. Over the course of six years, two separate initiatives to improve adequacy were implemented through the cooperation of members of the Departments of Pathology, Medicine and Diagnostic Radiology. This paper investigates whether these interventions, including restricting the performance of biopsies to subspecialist operators, provision of an adequacy statement in all renal biopsy reports and on-site adequacy assessments are effective in improving renal biopsy adequacy.

## Methods

### Ethical approval

Ethical approval derives from the *a priori* approval granted by the Research Ethics Board of the Capital District Health Authority to the Division of Anatomical Pathology to use aggregate de-identified patient data for quality improvement purposes.

### Setting

The Queen Elizabeth II Health Sciences Centre is a university-affiliated referral centre with an active renal transplant program that performs an average of 90 transplants per year. The anatomical pathology laboratory processes and reports approximately 200 native renal biopsies and 100 renal allograft biopsies per year, a number that has remained fairly stable over the last few years.

### Participants

An analysis of adequacy for percutaneous renal biopsies performed at our centre was conducted using aggregate de-identified patient data. Biopsies performed at other centers and intraoperative biopsies were excluded from the analysis.

### Interventions

The first intervention included:

1. Restriction of the performance of biopsies to a group of 11 radiologists from a group of approximately 20 radiologists with a subspecialty interest in this field of practice. No attempt was made to standardize biopsy technique among operators. All biopsies were performed with 16 gauge biopsy guns (C. R. Bard Inc., Murray Hill, New Jersey, USA) under real-time ultrasound guidance.
2. Provision of assessment of biopsy adequacy in every renal pathology report. Adequacy criteria for renal biopsies were locally defined through review of the literature, and discussion and consensus between the two renal pathologists who were practicing at the time that this was introduced. Two additional renal pathologists who participated in signing out renal biopsy reports during the course of the study also included the comment in their reports. It continues to be a standard statement in each renal biopsy report in our centre.

The second intervention was to have a medical laboratory technologist perform adequacy assessment on-site in the biopsy suite. The sample was examined under a dissecting microscope and the presence of glomeruli determined. If the sample was judged not to have glomeruli in any one of the cores for light, immunofluorescence or electron microscopy, then an additional core could be obtained. Three medical laboratory technologists were trained by pathologists assistants to identify glomeruli in renal biopsies examined under a dissecting microscope. The pathologists assistants had been trained previously by a renal pathologist. Any suboptimal or inadequate biopsies were discussed with the medical laboratory technologists after the renal biopsy report had been verified in an attempt to ascertain the reason why the particular renal biopsy was not adequate, such as a difficult procedure or patient factors.

### Definition of adequacy

#### Native renal biopsies

1. Adequate biopsies contained at least ten glomeruli for light microscopy and at least one glomerulus each for immunofluorescence and electron microscopy.
2. Biopsies that did not meet these criteria but contained at least one glomerulus each for light, immunofluorescence and electron microscopy were classified as sub-optimal.
3. Biopsies that failed to meet the sub-optimal criteria were classified as inadequate.

### Renal allograft biopsies

1. Adequate specimens contained ten or more glomeruli, two or more arteries, and at least one glomerulus each for immunofluorescence and electron microscopy.
2. Suboptimal specimens contained at least one glomerulus each for light, immunofluorescence and electron microscopy, and at least one artery, but did not meet the full adequacy criteria.
3. Allograft specimens that failed to meet the sub-optimal criteria were classified as inadequate.

### Data analysis

The percentages of adequate, suboptimal and inadequate native renal and allograft biopsies were calculated for three periods:

- Period 1: Baseline, October 2005 to September 2006 (12 months). Adequacy was determined retrospectively by a renal pathologist reviewing the renal biopsy report and applying the numerical adequacy criteria.
- Period 2: After implementation of the first initiative, January 2007 to September 2011 (57 months). For renal biopsies from this period an adequacy statement was included in the renal biopsy report by the renal pathologist by applying the numerical adequacy criteria. Renal biopsy adequacy was monitored by auditing the adequacy statement in renal biopsy reports every three months.
- Period 3: After implementation of the second initiative, October 2011 to September 2012 (12 months). Adequacy was determined as for period 2 above.

The differences in adequacy between periods were expressed as the change in percentage of adequacy in the period in question compared to the preceding period, i.e. period 2 compared to period 1, and period 3 compared to period 2, with 95% exact confidence intervals. The Fisher’s exact test was applied to determine the statistical significance of the changes between periods. A two-tailed probability was used with an alpha level of 0.05 used to determine significance.

After analyzing our results, in order to evaluate the relationship between the interventions and the improvement in adequacy, we questioned whether potential improvement was contributed to by an increase in the number of passes taken with the biopsy gun during renal biopsy collection. Since the number of tissue cores submitted would approximate the number of passes, we examined a subset of the native renal biopsies comparing the number of cores recorded in the renal biopsy report with adequacy for three consecutive three month periods:

- Subset period 1: Prior to implementing the interventions, July, August and September, 2006
- Subset period 2: Between implementing the first and second sets of interventions, July, August and September, 2011
- Subset period 3: After implementing the second set of interventions, July, August and September, 2012

The Fisher’s exact test was applied to determine the statistical significance of the difference in proportions of biopsies with greater than three cores between adequate and suboptimal or inadequate biopsies in each subset period.

Statistical calculations were performed using SAS version 9.4 (Cary, North Carolina, USA).

## Results

A total of 663 native renal biopsies and 339 allograft renal biopsies were included in our study.

During periods 2 and 3 the number of radiologists decreased from approximately 20 during the first period, to 11.

All reports during periods 2 and 3 contained an adequacy statement.

All renal biopsies during period 3 were assessed on site for the presence of glomeruli by one of three medical laboratory technologists.

The percentages of adequate, suboptimal and inadequate native renal biopsies during all three periods are listed in Table 1. The percentages of adequate biopsies for periods 1, 2 and 3 were 31%, 72% and 90%, respectively. This represents a significant increase (40%, 95% CI: 31% – 50%, p < 0.0001) in adequacy following the first initiative, and another significant increase (18%, 95% CI: 8% – 29%, p = <0.0001) following the second initiative.

**Table 1:** **Summary of adequacy assessment of native renal biopsies**

**Period**	**Total biopsies (n)**	**Adequate biopsies (n,%)**	**Suboptimal biopsies (n,%)**	**Inadequate biopsies (n,%)**	Difference in adequacy (%)*	**p value**
1	127	40 (31%)	43 (34%)	44 (35%)	NA	NA
2	432	311 (72%)	54 (13%)	67 (16%)	+40% (31, 50)	< 0.0001
3	104	94 (90%)	2 (2%)	8 (8%)	+18% (8, 29)	<0.0001

*Change of percentage of adequacy in the period in question compared to the preceding period, i.e. comparing period 2 to period 1, and comparing period 3 to period 2.

The percentages of adequate, suboptimal and inadequate allograft renal biopsies are listed in Table 2. The percentages of adequate biopsies for periods 1, 2 and 3 were 75%, 56% and 69%, respectively. This represents a decrease in adequacy that is not statistically significant (19%, 95% CI: 4% – 41%, p = 0.16) following the first initiative, and an increase that is not statistically significant (13%, 95% CI: 2% – 28%, p = 0.11) following the second initiative.

**Table 2:** **Summary of adequacy assessment of renal allograft biopsies**

**Period**	**Total biopsies (n)**	**Adequate biopsies (n,%)**	**Suboptimal biopsies (n,%)**	**Inadequate biopsies (n,%)**	Difference in adequacy (%)*	**p value**
1	20	15 (75%)	0 (0%)	5 (25%)	NA	NA
2	270	152 (56%)	81 (30%)	37 (14%)	−19% (4, 41)	= 0.16
3	49	34 (69%)	9 (18%)	6 (12%)	+13% (2, 28)	= 0.11

*Change of percentage of adequacy in the period in question compared to the preceding period, i.e. comparing period 2 to period 1, and comparing period 3 to period 2.

The subset of native renal biopsies analyzed for adequacy and number of cores comprised a total of 79 biopsies. Percentage of adequate biopsies by subset period; and number and percentage of adequate biopsies comprising more than three cores, compared to suboptimal and inadequate biopsies comprising more than three cores are listed in Table 3. Percentages of adequate biopsies for subset periods 1, 2 and 3 were 37%, 81% and 95% respectively showing the same gradual increase in adequacy after each of the interventions as in the larger data set. Adequate biopsies were less likely than suboptimal or inadequate biopsies to comprise more than three cores during subset period 1, while the opposite was true in subset periods 2 and 3, suggesting that one factor in adequacy may be an increase in core number, but these trends were not statistically significant.

**Table 3:** **Summary of adequacy assessment of subset of native renal biopsies, comparing number and percentage of adequate biopsies comprising more than three cores, to suboptimal and inadequate biopsies comprising more than three cores**

**Period**	**Total biopsies (n)**	**Adequacy in subset (%)**	**Number (%) adequate biopsies comprising cores >3**	**Number (%) suboptimal and inadequate biopsies comprising cores >3**	p- value*
1	27	37%	3 (30.0%)	10 (58.8%)	0.10
2	31	80%	10 (40.0%)	2 (33.3%)	1
3	21	95%	7 (26.9%)	1 (16.6%)	1

*Comparing number and percentage adequate of biopsies comprising more than three cores, to suboptimal and inadequate biopsies comprising more than three cores.

## Discussion

Our results show that restricting the performance of biopsies to subspecialist operators, including an adequacy statement in the renal biopsy report and on-site adequacy assessments were effective in significantly improving native renal biopsy adequacy. The combined effect of these two initiatives brought our centre’s renal biopsy adequacy to within the range reported in the literature [7-10]. Neither initiative was seen to result in a significant difference in allograft biopsy adequacy.

Analysis of a subset of native renal biopsies showed a trend of a greater number of biopsies comprising more than three cores in the adequate group as opposed to the suboptimal and inadequate group after each set of interventions, but the trend was not statistically significant. Factors such as awareness by radiologists of the issue, reducing the number of radiologists performing the procedure, therefore increasing their experience, feedback on performance to the operator though the adequacy statement in the report, enhanced by the physical presence of the medical laboratory technologist during the procedure may also play a role in improving renal biopsy adequacy.

One limitation of our study is that it did not assess the impact of the initiatives upon the incidence of post-procedure complications. Intuitively, one might expect that on-site adequacy assessment would increase the number of cores obtained at biopsy, and this in turn would result in an increase in complications. Manno et al. [11] observed no significant difference in complications in cases where a single tissue core was obtained compared to cases where multiple tissue cores were obtained in a prospective series of 417 biopsies. Patel et al. [12] point out that no previously reported series has demonstrated an increase in complications associated with obtaining more than one tissue core at biopsy.

The full adequacy criteria in our study for both native renal and allograft biopsies are more stringent than those found in most studies [2,4,5,7-17]. This is especially true in the case of native renal biopsies where adequacy has traditionally meant sufficient material to render a diagnosis. Partly as a consequence of this more inclusive standard, published adequacy percentages are high, between 89 and 100% [7-10]. However, there is support in the literature for the application of numerical criteria to native renal biopsies. The need for a minimum number of glomeruli to identify focal disease and to accurately classify disease activity has been demonstrated statistically [3,6]. Electron microscopy and immunofluorescence are often necessary to make a correct diagnosis, and the cases in which they are not necessary cannot be reliably predicted [2]. Consequently, we believe that our criteria are appropriate for assessing the diagnostic value of native renal biopsy specimens.

For allograft biopsies our adequacy percentages are within the range of those reported in the literature where the Banff 97/07 criteria were applied (55-95%) [12-17]. Even within this group of studies there is variation in the application of the Banff 97/07 adequacy criteria. Of note, the study with the highest reported adequacy used adequacy criteria of seven glomeruli and at least one artery, stating that the Banff 97 criteria stipulate that both adequate and suboptimal specimens indicate procedural success [12]. While this is true, we believe that stratification into fully adequate and suboptimal categories meaningfully reflects the diagnostic value of the specimen. Apart from these issues, the fact that both initiatives failed to significantly improve allograft biopsy adequacy is disappointing. We plan to investigate potential contributing factors as part of the ongoing quality improvement process for this important outcome.

### Limitations

Limitations include lack of the following:

1. Data on post-procedure complications, discussed above
2. Detailed analysis of the characteristics of the radiologists before and after the first intervention
3. Generally accepted and applied adequacy criteria for native and allograft renal biopsies in the literature to compare data between institutions impacting the generalizability our findings from a single centre to other institutions
4. Data to measure the impact of the second intervention independent of the ongoing impact of the first
5. Power to detect several sub-group differences

## Conclusion

Restricting the performance of biopsies to subspecialist operators, provision of a renal biopsy adequacy statement and on-site adequacy assessment are effective in significantly improving native renal biopsy adequacy. None of these changes results in a significant improvement in allograft biopsy adequacy. Therefore, increasing awareness of radiologists regarding adequacy, developing a pool of expert radiologists performing the procedure, providing feedback on performance to the operator through an adequacy statement in the report, and physical presence of the medical laboratory technologist during the procedure may improve native renal biopsy adequacy.
